# Success rates of probing for congenital nasolacrimal duct obstruction at various ages

**DOI:** 10.1186/s12886-020-01658-9

**Published:** 2020-10-08

**Authors:** Marta Świerczyńska, Ewelina Tobiczyk, Piotr Rodak, Dorota Barchanowska, Erita Filipek

**Affiliations:** 1 Departament of Pediatric Ophthalmology, School of Medicine in Katowice, Medical University in Katowice, Katowice, Poland; 2 Clinic of Pediatric Ophthalmology, prof. Kornel Gibiński University Clinic Centre, School of Medicine in Katowice, Medical University of Silesia, 35 Ceglana Street, 40-514 Katowice, Poland; 3 Students’ Scientific Society, Ophthalmology Clinic and Department of Ophthalmology, School of Medicine in Katowice, Medical University of Silesia in Katowice, Katowice, Poland

**Keywords:** Congenital nasolacrimal duct obstruction, CNLDO, Nasolacrimal duct probing, Epiphora

## Abstract

**Background:**

Although nasolacrimal duct probing is the standard treatment for congenital nasolacrimal duct obstruction (CNLDO) among children, the optimal timing of this procedure has been a topic of debate. The aim of the study was to analyze the clinical efficacy of nasolacrimal duct probing among patients with CNLDO symptoms at various ages.

**Methods:**

An 8-year retrospective study involved 2434 patients (3009 eyes), who underwent nasolacrimal duct probing conducted under topical anesthesia in the operating theatre. The study group consisted of 1148 girls (47.2%) and 1286 boys (52.8%) from 2 weeks to 41 months (average age was 8 ± 5.6 months). The participants were divided into nine age groups: 0–2 months, 3–6 months, 7–9 months, 10–12 months, 13–15 months, 16–18 months, 19–21 months, 22–24 months and over 24 months.

**Results:**

Bilateral obstruction was present among 575 (23.6%) children and was associated with a higher percentage of unsuccessful procedures compared to patients with unilateral obstruction (16.9% vs 10.2%, *p* < 0.001 Chi-square test). The success rate of the initial probing was 87.2% for all children and it was shown that it decreased with age. In the above age groups, it was 87.9%; 91.4%; 89.6%; 86%; 76.3%; 71.3%; 70.3%; 70.2%; 65.4%, respectively.

**Conclusions:**

Probing is a safe and effective procedure. However, age at the time of the initial intervention and bilateral surgery constitute significant risk factors for failed probing. Probing between 7 and 9 months appears to be reasonable treatment strategy for children without recurrent infections. Early surgical intervention may be considered for patients with additional signs.

## Background

Congenital nasolacrimal duct obstruction (CNLDO) is the most common cause of neonatal epiphora (excessive tearing) [1, 2]. It is a significant ophthalmological problem affecting approximately 11% of newborns [3], which is more common among Caucasian children born prematurely [3, 4]. The risk factors for this condition include the maternal infections, exposure to radiation, medications or some occupational hazards during pregnancy as well as a genetic predisposition [4–6]. The prevalence of CNLDO in Down syndrome has been reported to be 5–30% [6–8] and the bilateral obstruction is the most observed disease pattern among above children. Moreover, there are conflicting opinions on whether childbirth through cesarean section increases the chance of the occurrence of the CNLDO [3, 9–12]. The nasolacrimal duct is formed by canalization of the caudal extremity of an epithelial cord derived from the ectoderm in the naso-optic fissure. This process normally takes place at the end of 6 months of intrauterine life. However, it may be delayed for several weeks after birth. Obstruction is most often observed at the membrane of Hasner where the lacrimal duct empties into the nasal cavity [13].

Symptoms resulting from blocked outflow of tears may occur shortly after birth, but they appear more often and intensify in the following weeks due to the development of the tear production process [14]. The average time of diagnosis of obstruction is 5 weeks of age. In about 90% of cases, the condition is diagnosed by the primary care physician [3]. The most common symptoms include epiphora, tear stagnation, the presence of mucopurulent discharge in the conjunctival sac and on the eyelid margins, as well as crusting of the lashes. Also, when pressure is applied over the lacrimal sac, a reflux of mucoid or mucopurulent material from the punctum is observed. Sometimes, the residual debris undergoes a bacterial infection leading to purulent conjunctivitis [3, 15–20].

Persisting CNLDO, which occurs in 2–6% of all cases carries the risk of chronic dacryocystitis, preseptal and orbital cellulitis [10, 21]. Abundant lacrimal meniscus and mucopurulent discharge which persists on the surface of the cornea may lead to blurring of vision and interferes with the clear focusing of images on the retina, which is critical for emmetropization [22]. Several authors have confirmed an increased risk for anisometropia and amblyopia in patients with CNLDO (especially long-term untreated obstructions) [22–25]. However, the effect of long-term epiphora on visual development in children is debatable and some studies reported no significant differences between children with CNLDO and general population [17, 26, 27].

Spontaneous opening of the nasolacrimal duct is observed in 51.9–83.5% of children [1, 4, 9, 28, 29]. For this reason, conservative treatment is preferred during the first months after birth. In the event that the use of hydrostatic massage does not lead to the opening of the lacrimal ducts, nasolacrimal duct irrigating and probing is performed under general (GA) or topical anesthesia (TA). However, determining the optimal date to intervene has long been a subject of debate. Some researchers suggest early probing, and some other ophthalmologist prefers to perform this procedure later, after the first year of life.

The aim of the study is to present the results of the treatment of CNLDO by nasolacrimal duct probing and to evaluate the efficacy of the above procedure depending on the age of the child.

## Methods

### Study design

A retrospective analysis was carried out for treatments performed in children with symptoms of CNLDO who were treated at the Pediatric Ophthalmology Department of the Medical University of Silesia in Katowice from May 2012 to April 2020. The study was conducted in accordance with the tenets of Declaration of Helsinki and was approved by the Bioethics Committee of the Medical University of Silesia in Katowice. Written informed consent was obtained from the parents of each participant.

### Patients

**Fig. 1:**
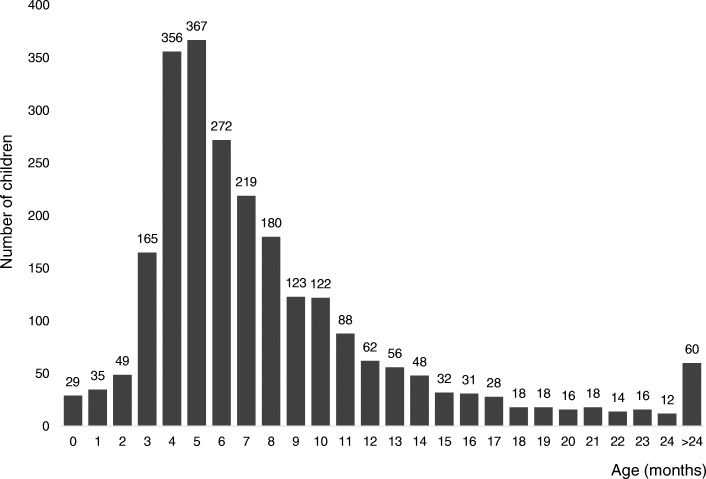
Number of children who underwent nasolacrimal duct probing according to age

The study included 3009 eyes in 2434 children aged from 2 weeks to 41 months, the mean age was 8 ± 5.6 months. Most were patients from 3 to 6 months of age (1160 children, 47.7% of subjects), and the most numerous group were 5-month-old patients (367 children, 15.1% of subjects) (Fig. 1). Procedures were performed in 1286 (52.8%) boys (average age 8 ± 5.7 months) and 1148 (47.2%) girls (average age 7.9 ± 5.4 months). Unilateral occlusion was present in 1859 children (76.4%), including 947 (38.9%) on the right side and 912 (37.5%) on the left side. Bilateral nasolacrimal duct obstruction was found in 575 patients (23.6%).

### Inclusion criteria

The diagnosis of CNLDO was based on the history of tearing and/or discharges of one or both eyes since or shortly after birth and confirmed by evidence of epiphora with or without mucopurulent discharge and regurgitation during examination. Before the procedure, the patients remained under the supervision of the Pediatric Ophthalmology Clinic. In cases where obstruction symptoms persisted, despite the use of conservative treatment (hydrostatic massage of the lacrimal sac area and antibacterial eye drops for the therapy of bacterial superinfections), children were qualified for surgical treatment. Parents were properly instructed to perform a correct Crigler maneuver 2–5 times a day in order to increase the chances of a CNLDO spontaneous resolution. Children under 6 months of age were eligible for the procedure if they were diagnosed with an abscess of the lacrimal sac, mucous cyst of the lacrimal sac or chronic purulent inflammation that persisted despite a proper conservative treatment. Patients were consulted by a pediatrician and anesthesiologist before the procedure. The probing was performed under TA in the operating theater under the supervision of an anesthesiologist. Among older children midazolam premedication was administered (dosage based on body weight and age) [30].

### Exclusion criteria

Patients were excluded from the study group in whom: previous procedures on the lacrimal ducts were performed; tearing was associated with other conditions (including facial malformations, eyelid position disorders, abnormal nasal bone structure, agenesis or ectopic lacrimal puncta, congenital fistulas of the lacrimal sack); and those who did not attend a follow-up visit to confirm the resolution of symptoms of nasolacrimal duct obstruction.

If there was no improvement after double probing, the children were referred for a laryngological consultation and diagnostic imaging and further management depended on the pathology found. In patients with no additional abnormalities found in the tear ducts, another, third probing was performed. Children who were diagnosed with complex obstruction during a detailed diagnosis and qualified for silicone intubation were excluded from the study.

### Treatment

In order to confirm the obstruction, the procedure of nasolacrimal duct probing was started by widening the superior lacrimal punctum and then irrigating the nasolacrimal ducts with a canula inserted through the upper lacrimal punctum. Probing was performed using a Bowman probe. Its diameter was selected adequately to the width of the lacrimal canaliculi, size ranging from size 00 (0,90 mm diameter) to size 1 (1,10 mm diameter). The next step was the introduction of a Bowman probe through the upper lacrimal punctum into the vertical duct perpendicular to the margin of the eyelid, later through the horizontal and common ducts, to the lacrimal sac. Moving the probe parallel to the margin of the eyelid, one can feel the hard contact with the medial wall of the lacrimal sac. Then the probe was turned upwards towards the brow bone and guided downwards by the nasolacrimal duct until it stopped – to the obstructed valve of Hasner. The obstruction site was passed through by a firm downward movement. After the Bowman probe was withdrawn, patency was checked by irrigating the lacrimal pathways with physiological saline. No complications were noted during or after the procedure. There were no cases of a false passage or injury to the nasolacrimal duct, canaliculi or puncta.

After the probing, application of a topical antibiotic was recommended from the fluoroquinolone group for 7 days in combination with hydrostatic massage [21]. Subjects had follow-up visits timed 3 weeks (± 1 week) and 6 months (± 1 month) after the procedure. Treatment was deemed successful once the 3 main clinical symptoms of CNLDO (epiphora, increased tear lake and mucous discharge) had subsided and result of dye disappearance test (DDT) was normal. DDT was performed by administering one drop of 2% fluorescein solution into the unanesthetized conjunctival fornices. After 5 min, each eye was evaluated for clearance with cobalt blue filter light of the slit lamp. Probing was successful when there was no fluorescence in the conjunctival sac or thin fluorescing marginal tear strip persisted. Unsuccessful probing was documented in case of presence of a wide, brightly fluorescing tear strip. If the initial probing was unsuccessful, repeated probing was performed on average 4 (from 1 to 6) months after it.

### Statistical analysis

Statistical analysis was carried out using the STATISTICA 13.3 software. The Shapiro-Wilk test was used to assess the occurrence of normal distribution among the studied variables. The quantitative variables analyzed did not meet the criterion of normality of distribution, and therefore the difference significance analysis was conducted using the non-parametric Mann-Whitney U test. The Chi-square test was used to assess the statistical significance of differences in the efficacy of the procedures among children with unilateral and bilateral obstruction. Logistic regression was used to compare the effectiveness of surgical treatment between individual age groups and to determine the effect of obstruction type and gender on the effectiveness of therapy. The results were evaluated in 95% confidence interval and the value of *p* < 0.05 was assumed as the level of statistical significance.

## Results

Nasolacrimal duct probing was performed as the first surgical intervention on 3009 eyes of 2434 patients with CNLDO symptoms. Among children under 6 months of age who were qualified for the procedure (1001 patients) an abscess of the lacrimal sac was diagnosed in 2,1%, mucous cyst of the lacrimal sac in 9,2% and chronic purulent inflammation persisting despite proper conservative treatment in 88,7% of them. In the study group, the success of the first treatment was recorded in 2624 eyes (87.2% of procedures). In contrast, 385 eyes (12.8% of procedures) required another procedure due to an unsatisfactory result. After the second probing, patency was obtained in 335 eyes (87% of procedures), and after the third, in the remaining 50 cases. The average age of children with the first successful probing was significantly lower than that of the patients in whom the above procedure was unsuccessful (7.6 ± 5.2 vs 11.1 ± 7.5 months, *p* < 0.001, Mann-Whitney U test).

**Fig. 2:**
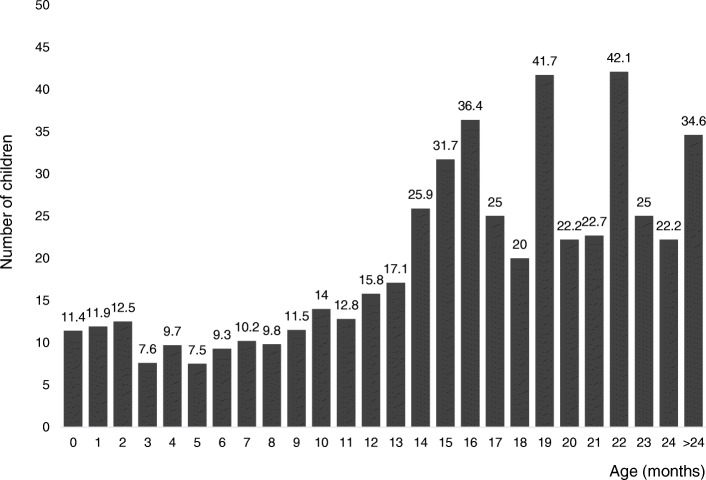
Failure procedures (%) according to age

Among the subjects aged from 3 months to 6 months, the number of failures in the first nasolacrimal duct probing did not exceed 9.7%, and the percentage of unsuccessful procedures was the lowest among 5-month-old patients and amounted to 7.5% (Fig. 2).

In the next age group (patients aged 7 months to 9 months) the failure rate was between 9,8% and 11,5%. In older age groups, in turn, the number of procedures requiring repetition was significantly higher (*p* < 0.001; Mann-Whitney U test). Attempts to obtain patency after 13 months of life were associated with almost 26% risk of failure. However, in children over 2 years of age, 1/3 of procedures did not yield satisfactory results.

In patients with bilateral nasolacrimal duct obstruction, a smaller number of successful surgeries was observed compared to children with unilateral obstruction (83.1% vs 89.8%, *p* < 0.001 Chi-square test) (Tab. 1). There were no statistically significant differences between the sexes.

**Table 1:** Results of multivariable logistic regression analyses evaluating factors predicting success of nasolacrimal probing

Variable	Eyes (*n* = 3009)	Successful probing n (%)	OR (95%CI)	*P* value
Age (months)				
≤ 2	141	124 (87.94)	0.69 (0.40–1.78)	0.1717
3–6	1430	1307 (91.4)	1	
7–9	634	568 (89.59)	0.81 (0.59–1.11)	0.1894
10–12	335	288 (85.97)	0.58 (0.40–0.83)	0.0027
13–15	169	129 (76.33)	0.30 (0.20–0.45)	< 0.0001
16–18	101	72 (71.29)	0.23 (0.15–0.37)	< 0.0001
19–21	64	45 (70.31)	0.22 (0.13–0.39)	< 0.0001
22–24	57	40 (70.18)	0.22 (0.12–0.40)	< 0.0001
> 24	78	51 (65.38)	0.17 (0.11–0.29)	< 0.0001
Type of obstruction				
Unilateral	1859	1668 (89.73)	1	
Bilateral	1150	956 (83.13)	0.56 (0.45–0.69)	< 0.0001
Gender				
Girls	1410	1239 (87.87)	1	
Boys	1599	1385 (86.62)	0.89 (0.72–1.11)	0.3036

*OR* Odds ratio, *CI* Confidence interval

## Discussion

Although the nasolacrimal duct probing is the standard therapeutic procedure used in children with CNLDO, the recommendations concerning the optimal time to intervene are divergent [12, 16, 18–20, 28, 29, 31–42]. Several authors have described an age-dependent decrease in the success rate of initial probing [12, 19, 29, 31, 32, 37] our study also confirm this correlation. The percentage of failure of the first probing was the lowest among patients aged 3–6 months (8,6%) and 7–9 months (10,4%). Subsequently the efficacy of this procedure decreased with the age. Similar results were also obtained by Grałek et al. [18] and Wójcik-Niklewska et al. [31].

Sathiamoorthi et al. found that in cohort of 1958 children, the period between 9 and 15 months of life was determined as the most appropriate for probing [29]. It was demonstrated that the chance of spontaneous opening of the nasolacrimal duct is the highest in the first 2 months of life (it amounts to approximately 85%), then it gradually decreases, and after the 9th month of life it reaches a plateau phase. Moreover, it was confirmed that probing after the 15th month of life is associated with a significantly lower percentage of successful procedures.

Moreover, based on cases of computed tomography facial series, Moscato et al. [35] indicated that the increases in height, volume and diameter of the nasolacrimal ducts occur mainly in the first 6 months of life. According to above study spontaneous resolution of CNLDO among infants may be linked to this anatomic evolution and surgical procedure before the age of 6 months should be avoided.

Some authors are in favor of performing the procedure later [20, 28, 33, 34, 36]. A prospective cohort study conducted by the Pediatric Eye Disease Investigator Group (PEDIG) did not show the relationship between the age at which the probing was performed and the success of this procedure up to 36 months of life [20]. In turn, some ophthalmologist postulate that nasolacrimal duct probing in both younger and older children is equally effective, and that the failure of the surgery does not depend on the age, but on the cause of obstruction [32, 34, 38, 39]. According to some retrospective studies, the percentage of unsuccessful procedures is greater in older age group due to a self-selection process. Medghalchi et al. [39] reported that the surgery success rate after 6 months was 91% among children with simple obstruction at the valve of Hasner versus 52% in patients with complicated types of obstruction. Kashkouli et al. [32] stated a success rate of late and very late probing was significantly lower in the complex (33.3%) than membranous (90.2%) group.

Arguably, the lower cure rate in patients probed later is not the result of age per se [39, 41]. Older children with CLDO may represent the pool of patients born with complex, non-valvular types of obstructions that did not resolve spontaneously in the first months of life and require different procedures, including nasolacrimal duct intubation, laser procedures and even dacryocystorhinostomy [41].

Bilateral obstruction, occurring in 9–47.5% [3, 4, 9, 11, 18–20, 23, 26–29, 31, 33, 34, 36, 38–40] may indicate a more complicated cause of the condition, create greater technical difficulties during procedures performed under local anesthesia, and thus affect the obtainment of less-satisfactory results, which is also confirmed by our study and reports by other researchers [20, 36]. Dietze et al. [43] suggest that patients with trisomy 21, allergic rhinitis/seasonal allergies, history of an upper respiratory tract infection within 1 month and obstructive sleep apnea may be correlated with a higher risk of failure with a probing.

Despite contradictory opinions, it should be borne in mind that the early implementation of surgical treatment prevents symptoms that are cumbersome for children and their parents. Early probing may lead to immediate resolution of symptoms, fewer physician visits and may then help to alleviate the anxiety for parents and irritability of the child caused by the inconvenience of persistent epiphora, discharge and recurrent infections [44]. Moreover, early correction avoids complications (such as acute or reccurent dacryocystitis, canaliculitis, preseptal and orbital cellulitis) and prevents months of morbidity due to epiphora and chronic dacryocystitis [19]. It has been noted that chronic or recurrent inflammation can lead to serious complications and fibrosis in the lacrimal drainage system that may reduce the efficacy of subsequent treatments. In addition, the frequent use of topical antibiotics during long-term conservative therapy conduces to drug resistance and ocular surface disorders [16, 45].

Probing in younger patients can be successfully performed under TA and the total cost of such procedure is even 10 times lower than under GA [46, 47]. It seems that that probing under TA is the less traumatic psychologically for the infant [40]. In addition, the US Food and Drug Administration issued a warning that exposure to common anesthetic agents over multiple procedures may impair brain development in children younger than 3 years [48].

Garrec et all [40]. calculated that the cost for conservative treatment of CNLDO is globally 1.56 more expensive than immediate probing (with the respect to only unilateral obstruction the conservative therapy is 1.89 more expensive than surgical procedure). The strategy which would apply the non-surgical treatment for patients ≤5 months of life and the probing to children > 5 months would be the most cost-efficient strategy.

According to the PEDIG, in a randomized trial of infants aged 6 to 10 months with unilateral CNLDO, the probing is likely more cost-effective than wait-and-see strategy [42].

We document high success rates of nasolacrimal duct probing between 3 and 9 months of age. The cure rate of above procedure decreases with increasing age and age over 13 months is a predictor of poor outcome. Based on the results presented in this study, as well as taking into account the clinical data presented above, it seems reasonable to perform this procedure between 7 and 9 months of age among patients with CNLDO without recurrent inflammations. Probing after 6 months of age can be considered for children with CNLDO and frequent, prolonged infections. Early surgical intervention (under 6 months of age) appears to be reasonable treatment strategy for patients with CLNDO and mucous cyst, lacrimal sac abscess, and significant dacryocystectasia with chronic purulent inflammation persisting despite proper conservative treatment.

The limitations of this study include its retrospective nature, single institution, non-standardized and incomplete medical documentation. In addition, the study group was a homogeneous population of Caucasian children which limits the possibility of comparing the results obtained by us to other populations. Additionally, there was no collected data regarding obstruction type as well as the type of the impression (“hard” of “soft” stop) during probing. Therefore, it was impossible to differentiate CNLDO into the simple and complex type. Simple CNLDO is defined as a membranous obstruction at the lower end of the nasolacrimal duct, interrupting of which is accompanied by minimal resistance. Complex CNLDO, in turn, can be further divided into the complete (probe cannot be passed into the nose) and incomplete (the probe can be eventually retrieved from the nose) type. Moreover, children who were diagnosed with complex obstruction during further diagnostics and then qualified for silicone intubation were excluded from the study. The analysis also did not include patients referred for further diagnostics after two unsuccessful probing and who did not come to our clinic for a follow-up visit with the results of the recommended tests. Taking into account the above limitations, our work presents the effect of age on the success rate of probing in different age groups. Further research is required, additionally taking into account the success rate in different type of obstruction in different age group.

## Conclusions

In summary, nasolacrimal duct probing is an effective procedure used in the treatment of children with CNLDO. However, there is still an open debate on the optimal timing for the implementation of surgical treatment. The final decision should be made after considering the balance of potential profit and loss account, remembering that the efficacy of this procedure decreases with the age of patients. It is crucial to consider the type and severity of the symptoms as well as the quality of life of the patient and the family. Probing between 7 and 9 months appears to be reasonable treatment strategy for children without recurrent infections. Early surgical intervention may be considered for patients with additional signs.

## Data Availability

The data that support the findings of this study are available on request from the corresponding author MŚ.

## Abbreviations

CNLDO: congenital nasolacrimal duct obstruction
DDT: dye disappearance test
GA: general anesthesia
TA: topical anesthesia
PEDIG: Pediatric Eye Disease Investigator Group
